# Probing microtubules polarity in mitotic spindles *in situ* using Interferometric Second Harmonic Generation Microscopy

**DOI:** 10.1038/s41598-017-06648-4

**Published:** 2017-07-28

**Authors:** S. Bancelin, C.-A. Couture, M. Pinsard, M. Rivard, P. Drapeau, F. Légaré

**Affiliations:** 1 Institut National de la Recherche Scientifique, Centre Energie Matériaux Télécommunications (INRS-EMT), Université du Québec, 1650 boulevard Lionel-Boulet, Varennes, QC J3X 1S2 Canada; 2 Département de Neurosciences, Centre de Recherche, Centre Hospitalier de l’Université de Montréal, Montreal (QC), 900 rue Saint-Denis, Pavillon R, H2X 0A9 Canada

## Abstract

The polarity of microtubules is thought to be involved in spindle assembly, cytokinesis or active molecular transport. However, its exact role remains poorly understood, mainly because of the challenge to measure microtubule polarity in intact cells. We report here the use of fast Interferometric Second Harmonic Generation microscopy to study the polarity of microtubules forming the mitotic spindles in a zebrafish embryo. This technique provides a powerful tool to study mitotic spindle formation and may be directly transferable for investigating the kinetics and function of microtubule polarity in other aspects of subcellular motility or in native tissues.

## Introduction

Over the years, Second Harmonic Generation (SHG) microscopy has emerged as an effective tool in biology^1–3^. Like in two-photon excited fluorescence (2P), this type of laser scanning microscopy is characterized by an intrinsic 3D sub-micron resolution that is robust upon light scattering and which allows for higher image depth when compared to confocal microscopy^4, 5^. SHG is a nonlinear optical process in which highly polarizable and non-centrosymmetric structures emit photons at exactly half the excitation wavelength^6^. Since SHG does not involve population transfer, it significantly reduces phototoxicity and is photobleaching-free^7^. The emitted light results from the coherent sum of the electromagnetic field generated by every single SHG emitter and thus scales quadratically with the number of aligned molecules sharing the same polarity^8^. Indeed, adjacent molecules of the same polarity will emit strong SHG signals due to constructive interference while the SHG signal will almost vanish in the case of adjacent molecules of opposite polarity^9^.

Over the years, many groups have demonstrated that exploiting the intrinsic nonlinear optical properties of the sample is a valuable, although challenging approach. In the case of SHG, since the signal is highly specific for dense non-centrosymmetric media^3^, only few biological molecules produce detectable SHG signals^1, 10–12^. SHG microscopy has been used to image collagen-rich tissues such as tendon^13^, cornea^14^, skin^15^, fascia^16^ and cartilage^17^. Furthermore, SHG signals have been obtained from the myosin band in skeletal muscles^1, 12^. Lastly, SHG signal have been reported in microtubules (MTs)^1, 11^.

MTs are a key component of the cell cytoskeleton, involved in structural support and intracellular transport. Formed as a lattice of tubulin heterodimers, having two different ends, MTs exhibit an intrinsic polarity. This polarity has important biological consequences since it determines the dynamics of polymerization and the directionality of molecular motor movement^18, 19^ and plays a role in the organization and function of various cell type^20–22^. The polarity of MTs has long been thought to be involved in the assembly of mitotic spindles^23^, responsible for the segregation of chromosomes during cell division. Indeed, near the centrosomes, the MTs exhibits the same polarity while in the center MTs are antiparallel^24, 25^. However, the relationship between MTs polarity and spindle dynamics during mitosis remains poorly understood, mainly because of the huge challenge to measure MTs polarity in spindles.

Several methods have been used to record the polarity of MTs. Currently, the gold standard remains the hook method^26^, based on electron microscopy to directly visualize individual MTs. However, this technique requires thin and fixed sections and has been so far limited to study few tissue preparations^27, 28^. Fluorescently labelled plus-end tracking proteins^29^ and laser ablation^30^ provide information about MT polarity, but the quantification remains indirect and these techniques are invasive. More recently, SHG microscopy has been proposed as an alternative method to probe, non-invasively, the polarity of MTs in thick living tissues. Individually, MTs are very weak SHG emitters. However, the constructive interference in MTs arrays that have the same polarity results in detectable signals that have been used to visualize MTs in neurons^31, 32^, cilia^12^, astroglial filaments^33^ and mitotic spindles^1, 34–36^. Kwan *et al*. demonstrated that the polarity of MTs can be extracted from the ratio of forward and backward SHG signals^32^ with a limitation in disordered MTs arrays, such as the mitotic spindle. Lastly, Yu *et al*. established a very elegant method to measure MT polarity in spindles, based on a combination of SHG and 2 P images of fluorescently labelled MTs^36^.

Here, we use interferometric SHG (I-SHG) to measure the polarity of MTs in mitotic spindles without requiring any exogenous labelling. Originally developed to characterize non-centrosymmetric crystals^37, 38^, I-SHG is based on the measurement of the phase of the SHG signal. In the past years, its potential for tissue imaging has been demonstrated with different proteins, such as myosin from skeletal muscle^39^ and collagen from tendon and cartilage^40, 41^. Having recently solved one of the main drawbacks of I-SHG^42^, namely the long imaging time, we demonstrate here the possibility to use I-SHG to record the dynamical evolution of MT polarity in mitotic spindles from live zebrafish embryos.

## Materials and Methods

### Experimental setup

I-SHG imaging was performed using a custom-built laser-scanning microscope (for a complete description of the setup see ref. 42). In short, the excitation source was a mode-locked Ti:Sapph laser (810 nm wavelength, 80 MHz repetition rate, ~150 fs pulses width, Tsunami, Spectra Physics) pumped by a 12 W Millenia Pro laser (Spectra Physics). Beam intensity was controlled using a half-waveplate and a Glan-Thompson polarizer. A 5 cm focal lens was used to focus the laser on a 20-µm BBO crystal (θ = 29.2°, Eskma Optics) to generate a reference SHG beam outside the microscope whose intensity was adjusted by moving the crystal closer or further from the focal point. Both the excitation (810 nm) and the reference SHG (405 nm) were collimated using a metallic spherical mirror. To pre-compensate the group delay introduced by the optical components downstream, two calcite wedges were inserted between two half-wave plates at 810 nm and a full-wave plate at 405 nm. A 1.5 mm thick BK7 glass plate was placed on a rotating mount to control the phase between the reference and the excitation beam. A half-wave plate (at 405 nm and 810 nm) was inserted in front of the scanner to control the incident polarization. The microscope incorporated two galvanometer-mounted mirrors (TillPhotonics GmbH), a telescope to overfill the back aperture of the z-motorized water-immersion objective (40x, NA 1.15, Olympus), appropriate spectral filters (two FF01-720/SP-25 and a FF01-405/10–25, Semrock) and a photomultiplier tube (R6357, Hamamatsu Photonics) set at 1050 V. Scanning and signal acquisition were synchronized using a custom-written LabView software and a multichannel I/O board (National Instruments). 50 × 50 µm^2^ images were recorded in the forward direction, in about 2 s, using 30 µs pixel dwell-time and typically 200 nm pixel size. The average power after the objective was typically 100–150 mW. Raw data visualization was performed with ImageJ (NIH, USA) and image processing was performed with MATLAB (The MathWorks) and Origin 10 (OriginLab) as described below.

### Phase extraction

I-SHG is based on the interference of the SHG with the reference beam to extract the phase of the signal generated in the sample (for a complete description of the method see refs 41 and 42). Briefly, the nonlinear relationship between the glass-window angle and the phase shift introduced was calibrated with a 350 µm thick y-cut quartz plate used as a sample. This allowed, when looking at biological samples, to introduce a control phase shift between the two SHG beams. Then, we acquired 9 images, with reference phase varying from 0° to 480° by 60° phase steps. Subtracting two-by-two the images, taken with a 180°-phase shift (0°–180°; 60°–240°; 120°–300°…), allowed us to extract the interferometric term and to interpolate both the amplitude and the relative phase following eq. 1:

1 $$I({\phi }_{ref})-I({\phi }_{ref}+\pi )=4\sqrt{{I}_{ref}{I}_{{\rm{samp}}}}\,\cos ({\phi }_{{\rm{samp}}}-{\phi }_{ref})$$

where φ_ref_ and I_ref_ (respectively φ_exp_ and I_exp_) stand for the phase and the intensity of the reference (respectively sample) SHG beams. Finally, the phase image was reconstructed by determining, in every pixel, the reference phase that corresponds to the maximum amplitude. Note that the rotation of the glass plate, used to scan the reference phase, imposed a dead-time between images and that the manual adjustment of the incident polarization and the depth of focus between phase-stack recording, to correct for the slight movement of the spindles result in about 45 s of temporal resolution.

### Phase correction

The use of two galvanometric mirrors to scan the incident angle of the excitation beam on the back pupil of the objective led to a change in the optical path, which is virtually equivalent to rotating the glass plate. Therefore, when scanning the laser to acquire images, the phase difference between the reference and the sample SHG is gradually shifted as we progress further from the center of the interferometric pattern. However, since this phase shift depends only on the excitation geometry, it is possible to calibrate this effect^42^. This was achieved using again a y-cut quartz plate as a sample to directly image the phase shift introduced in the microscope. Nevertheless, since the calcite wedges used to pre-compensate for the group delay dispersion brought by the microscope introduced the same correction in the whole field of view, we observed a sharp decrease in interferometric contrast as we progressed further from the center (see ref. 42 for further discussion of this effect). Therefore, it appeared that only a small portion (~100 × 100 µm^2^) of the field of view could be effectively used to perform I-SHG with laser scanning acquisition. While an important limitation to image biological tissues, this was sufficient to measure the phase of SHG signal from mitotic spindles.

### Sample preparation

Wild-type zebrafish (*Danio rerio*) of the TL line were bred and maintained according to standard procedures^43^. All experiments were performed in compliance with the guidelines of the Canadian Council for Animal Care and conducted at the *Centre de Recherche du Centre Hospitalier de l’Université de Montréal* (CRCHUM). All protocols were approved by the animal care committee of CR-CHUM (agreement number: N15018PMDz). Embryos were mounted in a 3 cm Petri dish with a 1 cm hole at the bottom sealed with a 170 µm-thick coverslip. Another coverslip was set on top to minimize sample motion during imaging. Temperature in the room was about 20 °C, which slows down the development compared to the standard developmental table^44^. Embryos were imaged within 4 h after fertilization.

## Results

**Figure 1:**
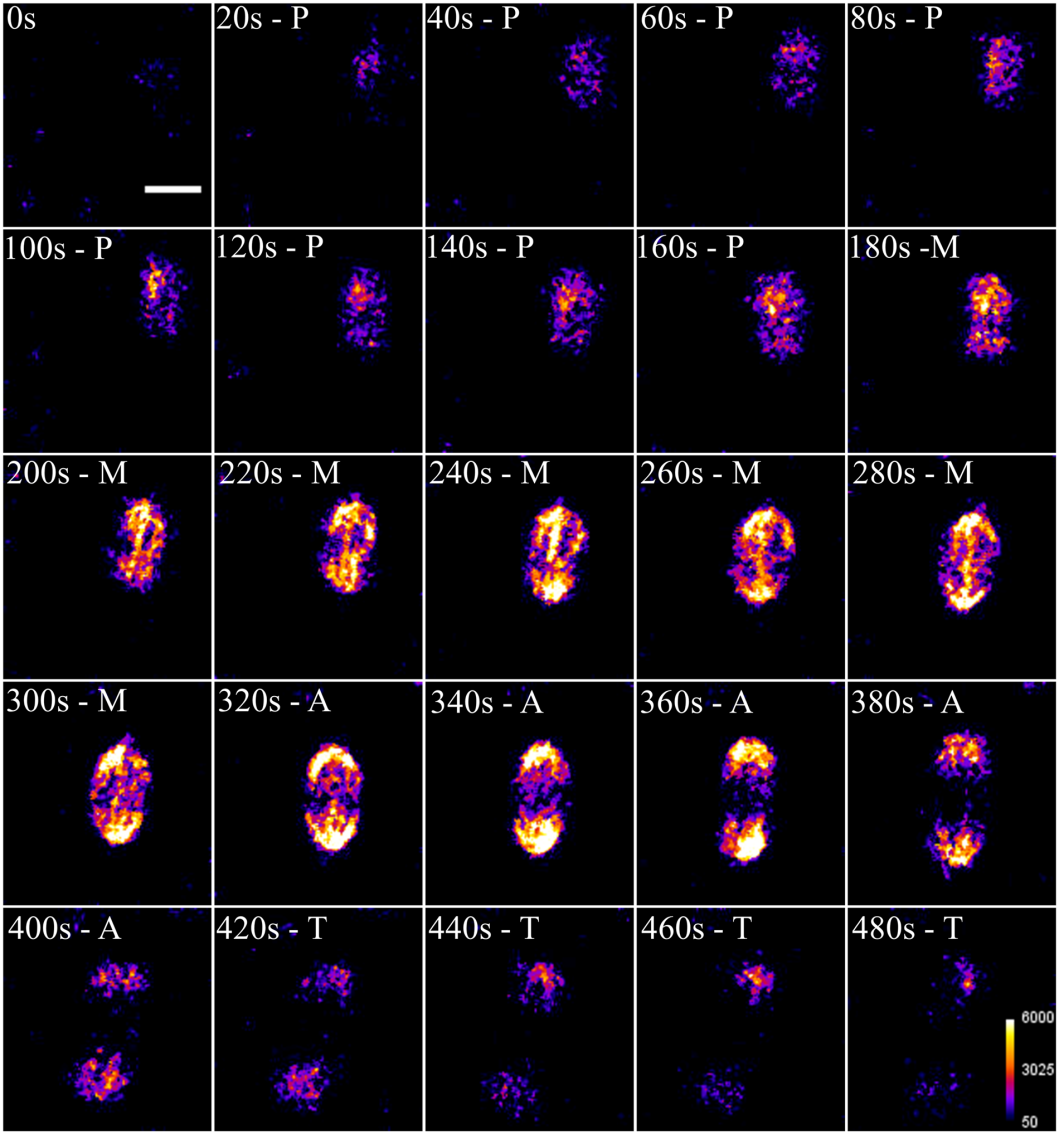
Time-lapse SHG imaging of mitotic spindles during mitotic division of a zebrafish embryo. Prometaphase (P; 20–160 s), metaphase (M; 180–300 s), anaphase (320–400 s) and telophase (420–480 s). Average power at focus: 150 mW, photomultiplier tube set at 1100 V. Scale bar: 10 µm.

Using time-lapse SHG microscopy, we imaged the mitotic divisions of a zebrafish embryo within 4 h after fertilization to record the dynamical behavior of mitotic spindles (Fig. 1). The SHG signal appeared during the prometaphase, when the MTs elongate to form the mitotic spindles (20–160 s). Then, the flattening and broadening of the MTs resulted in an increase in the SHG signal and revealed the alignment of the chromosomes between the centrosome during the metaphase (180–300 s). The migration of the chromosomes occurs in the anaphase (320–400 s) and SHG microscopy allowed us to clearly monitor the separation of mitotic spindles. Finally, the endogenous SHG signal gradually vanished during telophase while the mitotic spindles uncondensed and the MTs dispersed into the cell (420–480 s). Note that, during the metaphase, SHG from the middle region always appeared dimmer, as previously reported^1, 20^, which is due to an antiparallel alignment of MTs (see further in the Discussion).

**Figure 2:**
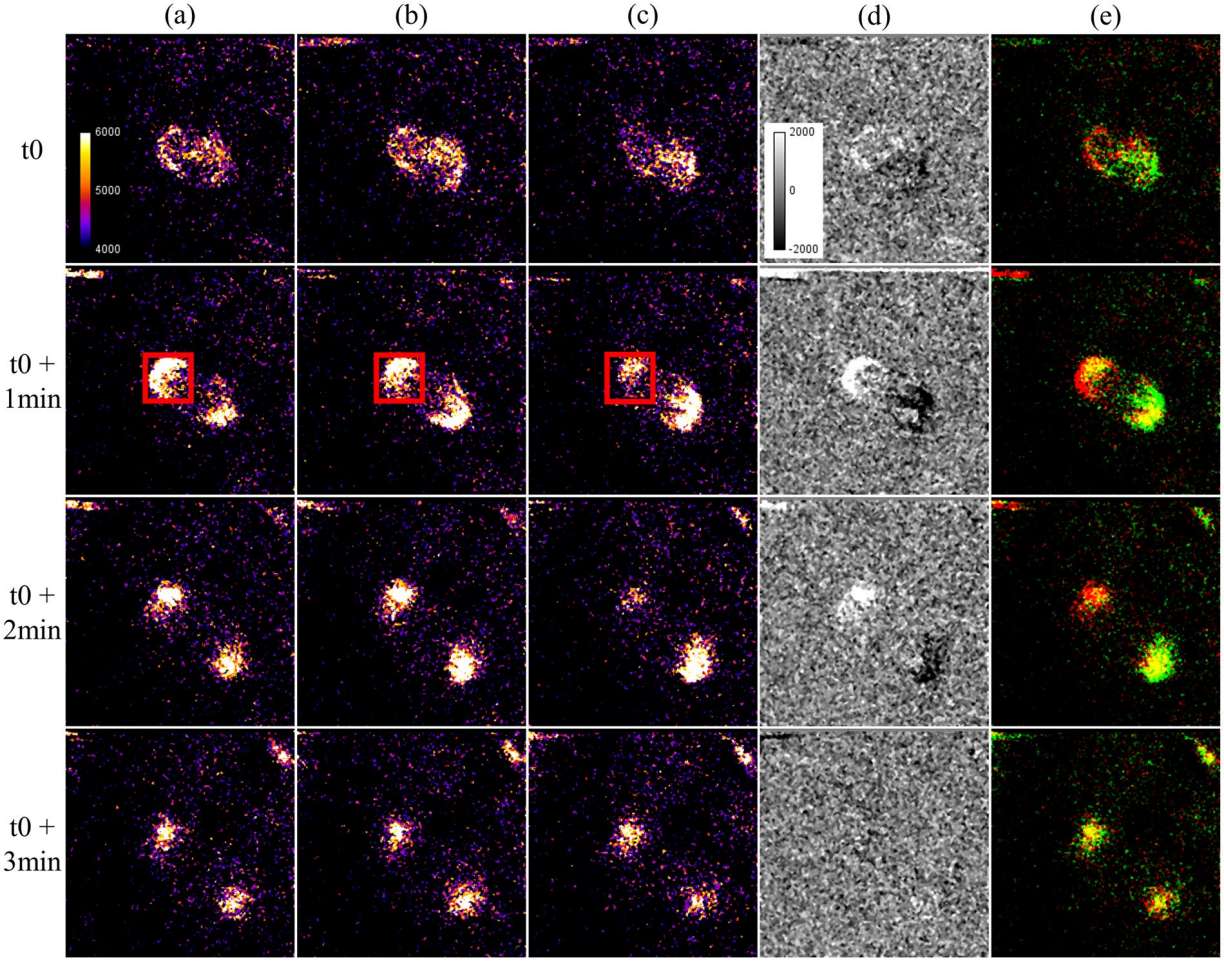
I-SHG of mitotic spindles in a zebrafish embryo. (**a**–**c**) Images acquired with different reference phases (0°, 90° and 180° respectively). Constructive and destructive interference occurs in opposite poles at 0° and 180° while no variations are observable at 90°. (**d**) Subtraction of the images (**a**,**c**) showing the interferometric contrast in the two poles. (**e**) 2-colors merge of the images acquired at 0° (in red) and 180° (in green). Average power at focus: 100 mW, photomultiplier tube set at 1050 V. Field of view: 50 × 50 µm^2^.

We then used I-SHG to visualize the change in spindle polarity throughout the course of mitosis. To that end, we acquired phase-stacks at two different times in metaphase and in anaphase (Fig. 2a–c). A median filter was applied to remove the noise. Note, the remaining background was not due to noise, but to the reference signal used in I-SHG. While varying the reference from 0° (a) to 180° (c), we could directly observe that the SHG intensity from pole varied. This is highlighted in the red rectangle, where the intensity was maximal at 0° of reference phase, due to constructive interference, diminished at 90° and was minimal at 180° since the reference and sample SHG interfered destructively. It is worth noting that the other pole exhibited the inverse behaviour, which highlights the interferometric nature of this variation.

To better visualize these variations of intensity, Fig. 2d and e display respectively the subtraction and the merged colors of the corresponding images (a and c). Notably, the variations of contrast upon the progress of mitosis in Fig. 2d indicates different degrees of polarity from the beginning of the metaphase to the end of anaphase. Furthermore, the different colors observed in the two poles in Fig. 2e revealed the presence of two opposite polarities. Note that no prior knowledge of the MTs orientation is required here since the optimization of the SHG intensity from the spindles (for a fixed reference phase) allows to match the laser polarization with the average MTs orientation.

**Figure 3:**
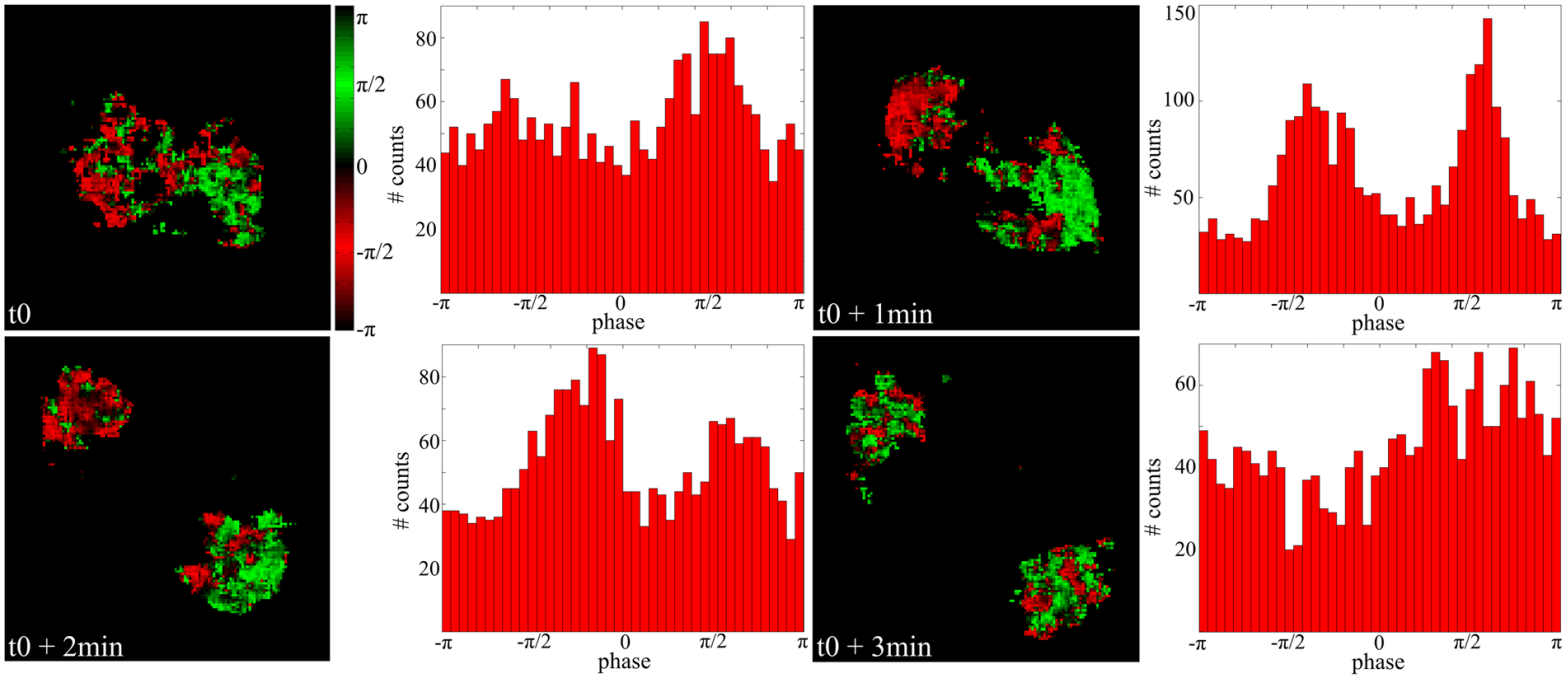
Image and histogram of the phase of the SHG signal generated in the spindles during mitosis. Field of view: 29 × 29 µm^2^.

From the reference phase stack, I-SHG allowed us to reconstruct the image of the phase in the sample and to extract the phase histogram (Fig. 3). The green and red pixels correspond to π-phase shifted signals, revealing opposite polarities of spindles. One can directly see that at the beginning of the metaphase (t_0_) the red and green pixels started to segregate, while the phase histogram showed two broad peaks, revealing the opposite polarities in the two poles. Then, at the end of metaphase (t_0_ + 1 min) and the beginning of the anaphase (t_0_ + 2 min), the spindles clearly had opposite polarities in the two poles, and the histograms show two narrower peaks. Lastly, at the end of the anaphase (t_0_ + 3 min), when the spindle uncondensed and the MTs disperse, the polarity was almost random around the two poles and the phase histogram is almost flat.

## Discussion

**Figure 4:**
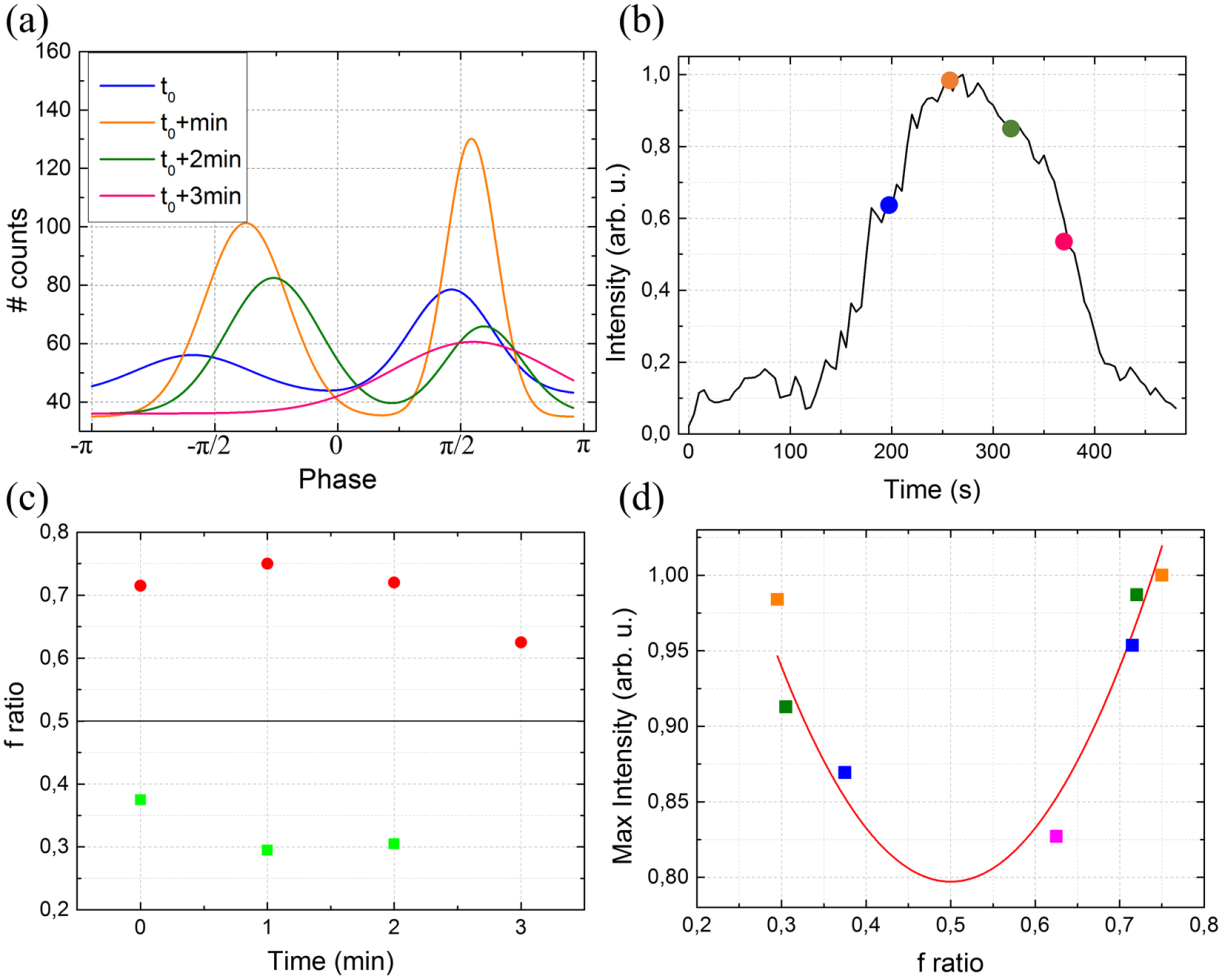
(**a**) Phase histogram at different time points highlighting the narrowing of the peak after 1 and 2 min. (**b**) Average intensity in the field of view (from Fig. 1) versus time. Colored circles correspond approximately to the time points in a. (**c**) Evolution of the f ratio with time. There is only one point at 3 min since only one peak (centered at π/2) is observable in the phase histogram at this time, due to quasi-random MTs polarity at this time. (**d**) Correlation between the maximum SHG intensity and the f ratio. The colored squared display the data extracted from (**a**) and the red line is the theoretical dependency (Eq. 3).

The SHG intensity is a good qualitative indicator of structural organization, since its quadratic dependency with oriented molecules^8^ makes it very sensitive of slight change in polarity. Yet, since SHG signals from MTs are rather weak and depend on a wide range of experimental parameters, quantification of structural changes based only on the signal intensity would be subjected to very large uncertainties. Figure 4a displays the superposition of the bi-Gaussian fitting of the phase histograms at different time points. At t_0_ + 1 min and t_0_ + 2 min, the phase distribution appears narrower, revealing a higher degree of polarity alignment of the MTs. In parallel, Fig. 4b shows the evolution of the average SHG intensity over time. Note that there is no reference signal in this case.

Altogether, Fig. 4a and b highlight the correlation between the SHG intensity and phase distribution. Indeed, almost flat phase distributions (t_0_ and t_0_ + 3 min) correspond to low SHG signal (around 200 s and 380 s), while peaked distributions (t_0_ + 1 min and t_0_ + 2 min) are associated with higher intensities (around 260 s and 320 s). As previously shown^40, 41^, one can quantify this effect, since the width of the phase peaks is related to the ratio of MTs with opposite polarities:

2 $$f=\frac{N(+{\beta }^{(2)})}{N(+{\beta }^{(2)})+N(-{\beta }^{(2)})}$$

where N(+*β* ^(2)^) and N(−*β* ^(2)^) are respectively the number of MTs with a positive and negative nonlinear susceptibility, or equivalently with one polarity or the other. Therefore, *f* = 0 or 1 when all the MTs are pointing in the same direction, while *f* = 0.5 when the MTs are randomly polarized in the focal volume. Note that there is an ambiguity in the definition of the *f* ratio, which reflects the ambiguity in the sign of the measured polarity. By convention, we attributed the *f* ratio below 0.5 to the negative phase peak (centered at −π/2) and the *f* ratio above 0.5 to the positive phase peak (centered at π/2). Determining the absolute sign of the polarity would require measuring the phase of the SHG signal in a reference sample.

Figure 4c displays the evolution of the *f* ratios calculated from the phase distribution over different time points. As expected, *f* is closer to 0.5 at the beginning of the metaphase (t_0_) and the end of anaphase (t_0_ + 3 min), since the MTs are arranged in a more disorganized array. In turn, at the end of the metaphase (t_0_ + 1 min) and the beginning of the anaphase (t_0_ + 2 min), *f* departs from 0.5, revealing the same polarity of MTs within highly aligned mitotic spindles.

The physics of SHG is well known^6^ and several studies have derived the theory of signal generation by a focused laser in biopolymers^8, 9, 36^. Assuming that all individual MTs generate equal signals, the SHG is directly proportional to the coherent sum of all the elementary responses. Thus, SHG intensity (I_SHG_) depends on intrinsic properties of the MTs as well as on their macromolecular arrangement (density and polarity):

3 $${I}_{SHG}\propto {|{\beta }^{(2)}|}^{2}{N}^{2}\,{(f-0.5)}^{2}$$

where β^(2)^ is the hyperpolarizability and N the total number of MTs in the focal spot. This equation shows that the SHG signal scales quadratically with the excess number of MTs in one direction. Extracting the *f* ratio from the phase histogram (in Fig. 4a) while simultaneously measuring the SHG intensity (with the reference signal) stands to verify this correlation. As shown in Fig. 4d, we observed an excellent correlation between the experimental measurements (colored squares) and the theoretical quadratic dependency (Eq. 3). Note that since SHG signal scales with the square of f-0.5, it cannot discriminate between a perfectly antiparallel network of MTs (f = 0.5) and the simple absence of MT. However, even a slight asymmetry (f = 0.55) in the MTs polarity distribution allows to detect the phase of the signal^41^.

## Conclusion

Since SHG microscopy does not require external perturbation, such as fixation or exogenous labelling, it can be applied in native tissues. Therefore, I-SHG microscopy offers a direct and dynamical method to quantify the degree of polarity of MT arrays in cells. We report here its first use in live cell imaging and show that I-SHG allows measurement of the evolution of MT polarity in mitotic spindles during the first divisions of a zebrafish embryo. It has long been proposed that this polarity plays a role in many cell processes and behaviors such as force transmission, cell migration, protein localization and transport. However, up to now, it has remained highly challenging to image MTs polarity *in situ*, impeding a proper investigation of these effects. Note that this method is not limited to imaging mitotic spindles but should be directly transferable to offer new insights into MT polarity in other systems such as dendrites^11^ or neuronal processes^28^.
